# The Microbial Zoo in the *C. elegans* Intestine: Bacteria, Fungi and Viruses

**DOI:** 10.3390/v10020085

**Published:** 2018-02-14

**Authors:** Hongbing Jiang, David Wang

**Affiliations:** Departments of Molecular Microbiology and Pathology & Immunology, Washington University in St. Louis School of Medicine, St. Louis, MO 63110, USA; hongbingjiang@wustl.edu

**Keywords:** host–pathogen interaction, *C. elegans*, intestine, bacteria, fungi, viruses, microbiome, trans-kingdom interactions

## Abstract

*C. elegans* is an invaluable model organism that has been a driving force in many fundamental biological discoveries. However, it is only in the past two decades that it has been applied to host–pathogen interaction studies. These studies have been facilitated by the discoveries of natural microbes that infect *C. elegans*, including bacteria, fungi and viruses. Notably, many of these microbes share a common site of infection, the *C. elegans* intestine. Furthermore, the recent descriptions of a natural gut microbiota in *C. elegans* raise the possibility that this could be a novel model system for microbiome and trans-kingdom interaction studies. Here we review studies of *C. elegans* host–microbe interactions with a particular focus on the intestine.

## 1. Preface

*Caenorhabidtitis elegans* is a free living nematode found in soil, compost pits and rotting fruits. It was brought from the field to the lab by Sydney Brenner over 50 years ago [1] and has been used to address many fundamental biological questions. For example, the Caspase cell death pathway [2] and RNA interference [3] were first described in *C. elegans* and were later found to be conserved in many species including humans. There are many key features that have made *C. elegans* such a successful model including: A short lifecycle of approximately three days coupled to a hermaphroditic lifestyle that facilitates genetics; transparency of the body that enables live fluorescent imaging studies; a completely defined developmental cell lineage [4]; facile RNAi screening via readily available whole genome RNAi libraries; it was the first metazoan organism with a completely sequenced genome [5]; availability of many mutants and transgenic animals to the community via the *C. elegans* Genetics Center.

As a differentiated multi-cellular organism, *C. elegans* mimics many aspects of mammalian physiology. Of particular relevance to many host–pathogen interactions is the *C. elegans* intestine, which as in higher eukaryotes, is the route of exposure and entry of many pathogens. The *C. elegans* intestine consists of 20 non-renewable epithelial cells, which make up the majority of its total body mass during development through the young adult stage. A key similarity between the *C. elegans* and human intestine is the presence of polarized epithelial cells with microvilli that are structurally attached to a terminal web composed of actin and intermediate filaments underneath the apical membrane [6,7]. The *C. elegans* intestine functions not only to assimilate nutrients, but also has the added function of detoxifying metabolites and toxins, like the liver in humans. It also consists of the first line of defense to invading pathogenic microbes. The intestinal lumen consists of the second-largest surface area in contact with the environment, besides the outer surface cuticle of the *C. elegans* body.

Here, we review host–microbial interactions in *C. elegans* with a focus on those that occur in the intestine.

## 2. The *C. elegans* Microbiome

In nature *C. elegans* lives in a complex environment, feeding on bacteria and fungi that are present in soil, compost and rotting fruits. Given this high degree of exposure to microbes, it is likely that in the wild, the *C. elegans* intestine may be populated by many different micro-organisms. In one study, 18 species of bacteria were identified in the microbiota of *C. elegans* that had been fed on soil and rotting fruits [8]. Furthermore, they found that the natural microbiota conferred protection from pathogenic *P. aeruginosa* infection. In 2016, three groups published papers characterizing the natural microbiome of *C. elegans* and found very similar habiting bacteria species in the *C. elegans* intestine from geographically different samples over the world [9,10,11,12]. The natural microbiome studied was shown to improve *C. elegans* growth, resistance to stress and relief from pathogenic bacteria and fungi infections. These observations parallel recent results in the mouse, demonstrating that “wild” microbiota provide a fitness advantage and increased resistance to environmental and infectious insults [13].

## 3. Bacteria–Host Interactions in the *C. elegans* Gut

**Figure 1:**
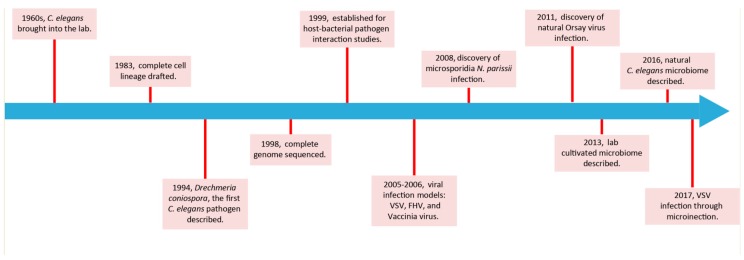
Milestones relevant to host-pathogen interaction studies in *C. elegans*.

Since most of the earliest studies of pathogen–host interactions in the *C. elegans* model focused on bacteria, it is natural that there are many reviews on this topic (Figure 1) [7,14,15,16,17,18]. We will focus specifically on the intestinal response of *C. elegans* to pathogen infections (Table 1).

In general terms, there are two major, non-mutually exclusive, mechanisms of bacterial pathogenesis in *C. elegans*. One is through secretion of toxins and the other is through direct colonization of the *C. elegans* gut lumen. Examples of both of these mechanisms are illustrated by studies using the well-established *Pseudomonas aeruginosa*-*C. elegans* infection model. The “fast killing” mode is mediated by the bacterial toxin when it is grown on high osmolality rich medium while a “slow killing” mode is observed when bacteria colonize the intestinal lumen [19]. Many bacteria produce or secrete different kinds of toxins to interact with the host that can cause pathophysiological changes to the host. For example, the pore forming toxins produced by *Baccillus thyrogensis* can perforate the *C. elegans* intestinal membrane, causing intestinal distention and animal death [53,54]. *Yersinia* bacteria encode an insecticidal toxin in a gene, *tca*A, which is genetically required for its toxicity to *C. elegans* [55]. *Burkholderia* also produce toxins to kill *C. elegans* [56,57]. *Enterococcus faecalis* express cytolysin that will lyse the host cells of infected *C. elegans* [58]. *Serratia marcescens* infection is also thought to kill by a toxin, suggested by genetic studies to be hemolysin [24]. Studies using *Staphylococcus aureus* showed that secreted α-hemolysin can lyse *C. elegans* cells just as it can lyse mammalian cells [25,26]. *Streptococcus* *pyogenes* and other *Streptococcus* species can synthesize hydrogen peroxide which is sufficient to kill the infected *C. elegans* [27]. Many of these toxin producing bacteria also exert pathogenic effects on *C. elegans* via colonization of the intestinal lumen. By contrast, *Listeria monocytogenes* does not appear to produce any toxins and causes *C. elegans* death exclusively through its accumulation in the *C. elegans* intestine [59].

**Figure 2:**
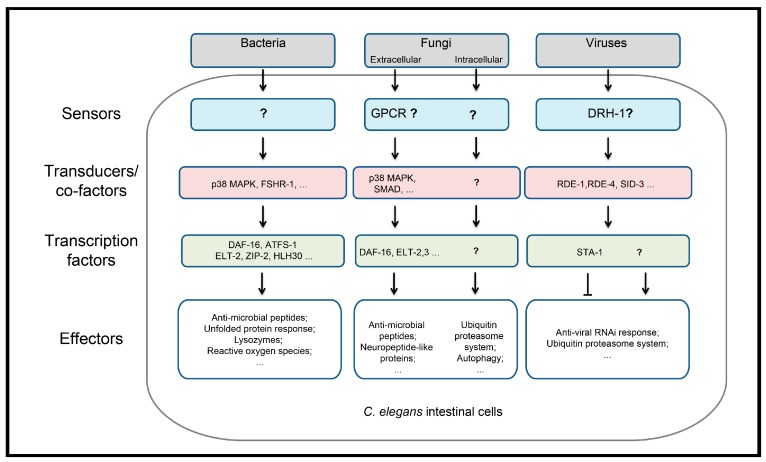
Known host genes that interact with bacterial, fungi and viruses in *C. elegans* intestinal cells. MAPK: Mitogen-Activated Protein Kinase; FSHR: Follicle Stimulating Hormone Receptor; DAF: Dauer Formation abnormal; ATFS: Activating Transcription Factor associated with Stress; ELT: Erythroid-Like Transcription factor; ZIP: bZIP (basic Leucine Zipper) domain; HLH: Helix Loop Helix; GPCR: G-Protein-Coupled Receptor; SMAD: Sma and Mad proteins from *Caenorhabditis elegans* and *Drosophila*; DRH: Dicer Related Helicase; RDE: RNAi Defective; SID: Systemic RNA Interference Defective; STA: Signal Transducer and Activator.

In the face of these pathogenic bacteria, *C. elegans*, like all hosts, has evolved multiple layers of defenses. At the organismal level, a behavioral response to avoid specific bacterial pathogens has been described [60,61]. In addition, *C. elegans* has physical defenses such as the pharynx grinder that disrupts most bacterial and the intestinal membrane that serves as a barrier to infection [62]. There are also innate immune defense mechanisms that are induced to counteract bacterial infections (model in Figure 2). *C. elegans* expresses different antimicrobial peptides, caenopores, lysozymes, lectins and reactive oxygen species as effector molecules to either detoxify the bacteria toxins or directly kill the invading bacteria (reviewed in [16,63]). A central question that has attracted significant scrutiny, but remains elusive, is the identity of the *C. elegans* pattern recognition receptor(s) (PRR) responsible for detecting bacterial infection. C-type lectins and chemoreceptors were proposed to be *C. elegans* pathogen recognition receptors based on their importance in recognition of pathogen associated molecular patterns (PAMPs) in other organisms [64,65]. It is also possible that detection relies on sensing perturbations of core host processes (also referred to as effector triggered immunity or damage associated molecular patterns (DAMPs)) induced by bacterial toxins [66]. In contrast to the uncertainties associated with the pathogen recognition step, the signaling cascades downstream of recognition have been extensively characterized. The canonical p38 PMK-1 (P38 Map Kinase-1) MAP (Mitogen-Activated Protein) kinase pathway is critical in *C. elegans* for defense against many bacterial infections as well as eukaryotic pathogens [20,23,67,68,69,70]. Other pathways including the TGFβ/DBL-1 (Transforming Growth Factor β/Decapentaplegic/Bone morphogenic protein like-1) and insulin signaling/DAF-2 (Dauer Formation abnormal-2) pathways have also been reported to be involved in the response to bacterial infections [71]. The G-protein coupled lectin like receptor FSHR-1 (Follicle Stimulating Hormone Receptor-1) was shown to be required for immune response during *P. aeruginosa* infection in *C. elegans* intestine [21]. A number of key transcription factors that regulate bacterial pathogen response have been reported, including the ZIP-2/ATFS-1 (bZIP transcription factor-2/Activating Transcription Factor associated with Stress-1) bZIP (Basic Leucine Zipper) transcription factors [22,72], DAF-16 FOXPO (Forkhead box protein O) transcription factors [73], GATA transcription factor ELT-2 (Erythroid-Like Transcription factor-2) [74] and the TFEB/HLH-30 (Transcription factor EB/Helix Loop Helix-30) transcription factors [75].

## 4. Fungi–Host Interactions in the *C. elegans* Gut

*N. parisii*, a natural microsporidial pathogen of *C. elegans*, was discovered in 2008 [6]. *N. parisii* was the first intracellular pathogen of the *C. elegans* intestine to be identified, in contrast to the previously studied bacterial pathogens which colonize in the intestinal lumen but generally do not infect the intestinal cells. Microsporidia infection significantly alters the *C. elegans* intestinal structure [6,28,29]. During the microsporidia lifecycle, meronts form inside the infected *C. elegans* intestinal cells and then exit the infected cells non-lytically by remodeling the intestinal terminal web [30]. In analyzing the transcriptional response of *C. elegans* to *N. parisii* infection, multiple Skr-Cullin-F-box protein ubiquitin ligase components were found to be highly upregulated. The cullin *cul-6* and Skp-1-related genes, *skr-3, 4, 5* were found to be important for ubiquitin targeting of *N. parisii* to the autophagy and proteasome degradation pathways [29].

The opportunistic human pathogen *Candida albicans* can infect the *C. elegans* intestine. Pukkila-Worley et al. established a model infection system in *C. elegans* to understand the host factors that control susceptibility to candidiasis [31]. They found that the yeast form of *C. albicans* can infect intestinal cells and cause distal intestinal distention. By contrast, heat killed *C. albicans* are avirulent, suggesting that the pathogenesis is not mediated by toxins. Analysis of the transcriptional response demonstrated that both live and heat killed *C. albicans* induced a similar response suggesting the host response is meditated by recognition of a fungal motif that is not dependent on growth. As the host response to *C. albicans* infection showed minimal overlap with the responses to pathogenic bacteria such as *S. aureus* and *P. aeruginosa* [31], it appears that *C. elegans* can distinguish between the invading pathogens and mount distinct transcriptional responses to different pathogens.

In contrast to what is observed with Candida, both live and heat killed *Cryptococcus neoformans* are lethal for *C. elegans*, suggesting that the pathogen kills the host by production of virulence factors [32,33].

Other natural fungal pathogens of *C. elegans*, such as *Drechmeria coniospora*, that infect the cuticle rather than the intestine have also been studied [76]. A host G-protein coupled receptor, DCAR-1 (Dihydrocaffeic Acid Receptor-1), was found to regulate the antifungal response by binding to an endogenous ligand [77]; the classic p-38 MAP Kinase PMK-1 pathway is implicated in downstream steps.

## 5. Virus–Host Interactions in the *C. elegans* Gut

From its initial establishment as a model until earlier this decade, there were no known viruses that could naturally infect *C. elegans*. Therefore, the first studies of virus–host interactions in *C. elegans* relied upon surrogate systems that included analysis of *C. elegans* cells, artificial infection conditions, or replicons [78,79]. Specifically, these entailed studies of vesicular stomatitis virus (VSV) infection of primary *C. elegans* cell culture [34,35], artificial virus infection in *C. elegans* using vaccinia virus [37], and a flock house virus transgenic system [38] (Table 1).

In 2005, two groups published papers using VSV infection of *C. elegans* primary cells. GFP (Green Fluorescent Protein)-encoding strains of VSV virus replicated to higher levels in cells derived from the RNAi deficient mutant *rde-1* and *rde-4* while the replication was inhibited in cells derived from mutants exhibiting enhanced RNAi responses, such as *rrf-3* and *eri-1*, demonstrating that RNAi is antiviral in *C. elegans* [34,35]. In parallel, a flock house virus replicon system was established that similarly identified RNAi as an antiviral pathway in *C. elegans* [38]. The Flock house virus B2 protein acts as an RNAi antagonist by binding double stranded RNA which then prevents Argonaute protein binding [39]. Lu et al. found that the flock house transgenic virus replication in the N2 wild type *C. elegans* was inhibited when the B2 protein was removed from the transgenic system, but replication of this mutant was restored in the *rde-1* RNAi deficient mutant [38,40]. A subsequent RNAi screen identified host factors in the RNAi pathway that are important for the control of virus replication, such as *rde-1*, *rde-4*, *dcr-1*, *rsd-2* and *drh-1* [40]. Both of these approaches were innovative and provided novel insights into the role of RNAi in *C. elegans*; however, there are also limitations to both. For the former, the cumbersome nature of isolating and maintaining embryonic cells [80,81] has precluded widespread use of the primary cell system to interrogate host–virus interactions in *C. elegans*. For the latter approach, replicon systems cannot be used to study multiple aspects of the viral lifecycle, such as viral assembly, egress or transmission.

As an alternative approach, polyethylene glycol (PEG) treatment of *C. elegans* enabled vaccinia virus to enter and then replicate [37]. Mutants in the cell death pathway lacking *ced-3* and *ced-4* were found to be more susceptible to vaccinia virus infection. This study highlighted a non-RNAi pathway that acts to restrict virus infection in *C. elegans*. Interestingly, when using a vaccinia virus that expresses β-galactosidase, the authors found blue X-gal staining in intestine in all infected animals, suggesting that the intestine is the primary site of infection in this model. To date no additional studies have been published using this system.

The first natural virus of *C. elegans* was not discovered until 2011 [41]. Through field sampling of wild nematodes and next-generation sequencing (NGS), three *Caenorhabditis* nematode viruses, Orsay virus, Santeuil virus, and Le Blanc virus, were isolated and identified [41,42]. All three viruses specifically infect intestinal cells [43] and share similar morphological disease symptoms that primarily affected the intestine including convolution of the intestine, loss of gut granules and fusion of intestinal cells [41]. Orsay virus can infect *C. elegans* strains, including the laboratory N2 strain, while Santeuil and Le Blanc virus infect wild *C. Briggsae* strains but not any tested *C. elegans* strains. To date, Orsay virus is still the only known virus that can naturally infect *C. elegans*. Genome characterization showed that these viruses share some similarity to nodaviruses [44]. RNAi pathway mutants such as *rde-1*, *rde-4* and *drh-1*, supported about 100-fold more virus replication than the wild type N2 strain, unambiguously demonstrating that in *C. elegans* RNAi plays a role in antiviral immunity against an authentic *C. elegans* viral pathogen [41,45,46]. While many viruses of plants and animals encode antagonists of the RNAi pathway, there is no evidence to date that Orsay virus encodes an RNAi antagonistic protein [47]. Subsequent studies found that the wild JU1580 *C. elegans* strain from which Orsay virus was initially isolated carries a deletion polymorphism in the *drh-1* gene which makes this strain highly virus-susceptible [45]. Interestingly DRH-1 (Dicer Related Helicase-1) is distantly related to the mammalian protein RIG-I (Retinoic Acid Inducible Gene-I), which plays a critical role in sensing of RNA viruses and subsequently triggering antiviral interferon induction. It appears that DRH-1 shares a conserved function in sensing intracellular viral RNA, but rather than inducing interferon or an interferon-like gene in *C. elegans*, DRH-1 is critical for antiviral RNAi activity. In fact, domain swapping experiments demonstrated that the helicase domain and C-terminal regulatory domain (CTD) of RIG-I could functionally substitute for the corresponding DRH-1 domain in *C. elegans* [46]. Independent of RNAi, a recent genetic screen identified a novel antiviral mechanism that requires CDE-1 (Caffeine Induced Death-1), a terminal uridylyltransferase [48].

Multiple studies have defined the transcriptional response to Orsay virus infection [29,49,50]. Given that Orsay infection is limited to the intestinal cells, these results most likely represent transcriptional changes within the intestine. A particularly noteworthy aspect of these studies is that Orsay virus infection and microsporidial infection by *N. parisii* induced a shared set of genes [29,50]. The *cul-6*, *skr-3* dependent ubiquitin pathway was found to be induced by both pathogens. RNAi knockdown of *cul-6* led to increased Orsay virus infection [29]. In addition, by further comparing the responses of *C. briggsae* to Le Blanc and Santeuil virus infection, a set of evolutionarily conserved nematode genes that respond to virus infection was defined [50]. Intriguingly, the functions of the majority of these genes are currently unknown, so it is not clear whether these are antiviral genes or genes necessary for viral (and potentially microsporidial) proliferation. In this set, multiple paralogs in the *pals* gene family [82], which has undergone significant expansion in *C. elegans* [83], were highly upregulated. While no clear function for any of these genes in relationship to virus infection has been defined, some of these genes have recently been implicated in regulating protein homeostasis [84].

In an effort to define the regulation of the transcriptional response to Orsay virus infection, promoter motif finding yielded a consensus resembling that of the STA-1 (Signal Transducer and Activator-1) transcription factor [51]. STA-1 is orthologous to mammalian STAT1 (Signal Transducer and Activator of Transcription1), which is a key signaling intermediate essential for interferon signaling in mammals. Mutant *C. elegans* lacking STA-1 were more resistant to Orsay infection than WT animals, suggesting that STA-1 acts as a negative regulator of the *C. elegans* antiviral response, in contrast to the positive regulatory role of the mammalian STAT1. Because STAT1 is known to be regulated by phosphorylation, a targeted kinase RNAi screen in *C. elegans* was pursued that identified SID-3 (Systemic RNA Interference Defective-3), a non-receptor tyrosine kinase [85], as a candidate regulator of STA-1 [51]. Strikingly, *sid-3* was simultaneously identified as a gene required for Orsay virus infection through an independent genetic screen [52]. That study also identified another gene, *viro-2*, which is orthologous to human Wiskott Aldrich Syndrome proteins (WASP), as being required for Orsay virus infection. Both of these genes were required for an early stage of Orsay virus infection; thus these genes may play key roles in entry of Orsay virus from the intestinal lumen.

Recently, direct VSV virus particle injection into *C. elegans* animals was demonstrated to lead to virus replication [36], raising the possibility that this strategy could be used to study a wide range of viruses in *C. elegans*. In this study, unique tissue tropism for muscle was observed following VSV injection, demonstrating that virus infection of non-intestinal cells in *C. elegans* is possible. In addition, virus replication was observed in intestinal tissue in *drh-1* mutant background. A limitation of this strategy is the apparent inability of infected animals to transmit infection to other animals, thus requiring direct microinjection of each individual animal to be analyzed.

## 6. *C. elegans* as a Model to Study Microbial Trans-Kingdom Interactions

To date, only a limited number of studies have attempted to directly study interactions of microbes from more than one kingdom in *C. elegans*. In one study infection by the fungal pathogen *C. albicans* suppressed anti-bacterial response in *C. elegans* [31], suggesting that Candida infected *C. elegans* may be more susceptible to bacterial superinfection. In another study, co-infection with *C. albicans* and many other gram-negative bacteria all showed that secondary infection of bacteria can inhibit the fungal growth [86,87]. With the discoveries of natural bacterial, fungal and viral pathogens of *C. elegans*, it is now possible to further explore potential trans-kingdom interactions. To date, there have been no studies that evaluated potential virus–fungi interactions or virus–bacterial interactions. For example, although it is known that both *N. parisii* and Orsay virus can infect intestinal cells, there have been no published reports evaluating potential interference or synergy in co-infection or superinfection models. Similarly, there are no reports comparing the impact of a “wild” microbiome vs standard OP50 *E. coli* on viral infection. Given the ease with which “germ-free” *C. elegans* can be generated by bleaching, coupled to the ability to readily control which bacterial species it is exposed to, *C. elegans* may be a very attractive model to define the function of the microbiome, and its interactions with the host and other fungal and viral agents.

## 7. Conclusions

*C. elegans*, as a simple eukaryotic model organism, provides a tremendous opportunity to define fundamental host–microbe interactions. Starting with the pioneering studies of bacterial pathogenesis, there have now been parallel efforts to use this model to elucidate evolutionarily conserved principles of fungal and viral pathogenesis. There is undoubtedly still much to be learned from these individual systems. However, the time is now ripe to also begin using this model to explore the more nuanced and complex world beyond mono-infection, in order to define the relationships and impacts of microbes on each other.

## Figures and Tables

**Table 1:** Pathogen–host interaction in the *C. elegans* intestine.

Kingdoms	Pathogens	Infection and Pathogenic Mode	Host Pathway and Response in the Intestine	Reference(s)
Bacteria	*P. aeruginosa*	Feeding; slow killing by colonization and fast killing by toxin	PMK-1, ZIP-2, FSHR-1 dependent pathways	[19,20,21,22]
	*S. enterica*	Feeding; killing by colonization; LPS as virulence factor	PMK-1-dependant programmed cell death pathway	[23]
	*S. marcescens*	Feeding; colonization causes distended intestine; LPS and hemolysin as virulence factors	DBL-1/TGF-β pathway	[24]
	*S. aureus*	Feeding; α-hemolysin as virulence factor	SEK-1 and NSY-1 dependent p38 MAP kinase pathway and TFEB mediated transcriptional response	[25,26]
	*S. pyogenes*	Feeding; colonization; hydrogen peroxide as virulence factor	Not analyzed	[27]
Fungi	*N. parisii*	Feeding; intracellular infection	CUL-6, SKR-3, 4, 5 ubiquitin ligase pathways	[6,28,29,30]
	*C. albicans*	Feeding; colonization;	PMK-1/p-38 MAPK pathways	[31]
	*C. neoformans*	Feeding; colonization; laccase; polysaccharide capsule and/or melanization as virulence factors	CED-1, C03F11.3 and ABL-1 dependent pathways	[32,33]
Viruses	VSV	Infection of primary cell culture or microinjection	RNA interference	[34,35,36]
	Vaccinia virus	PEG permeabilization	CED-3 and CED-4 Cell death pathways	[37]
	Flock house virus	Transgenic initiation of virus replication	RNA interference	[38,39,40]
	Orsay virus	Feeding	RNA interference CUL-6 ubiquitin-proteasome degradation; STA-1 repression and CDE-1	[41,42,43,44,45,46,47,48,49,50,51,52]

PMK: P38 Map Kinase; ZIP: bZIP (basic Leucine Zipper) domain; FSHR: Follicle Stimulating Hormone Receptor; LPS: Lipopolysaccharide; DBL: Decapentaplegic/Bone morphogenic protein like; TGF: Transforming Growth Factor; SEK: SAPK/ERK Kinase; NSY: Neuronal Symmetry; MAP: Mitogen-Activated Protein; TFEB: Transcription factor EB; CUL: Cullin; SKR: Skp1 Related; CED: Cell death; ABL: Abelson Murine Leukemia; VSV: Vesicular Stomatitis Virus; PEG: Polyethylene Glycol; STA: Signal Transducer and Activator; CDE: Caffeine Induced Death.

## References

[1] Brenner S. (1974). The genetics of *Caenorhabditis elegans*. Genetics.

[2] Ellis H.M., Horvitz H.R. (1986). Genetic control of programmed cell death in the nematode *C. elegans*. Cell.

[3] Fire A., Xu S., Montgomery M.K., Kostas S.A., Driver S.E., Mello C.C. (1998). Potent and specific genetic interference by double-stranded RNA in *Caenorhabditis elegans*. Nature.

[4] Sulston J.E., Schierenberg E., White J.G., Thomson J.N. (1983). The embryonic cell lineage of the nematode *Caenorhabditis elegans*. Dev. Biol..

[5] *C. elegans* Sequencing Consortium (1998). Genome sequence of the nematode *C. elegans*: A platform for investigating biology. Science.

[6] Troemel E.R., Felix M.A., Whiteman N.K., Barriere A., Ausubel F.M. (2008). Microsporidia are natural intracellular parasites of the nematode *Caenorhabditis elegans*. PLoS Biol..

[7] Pukkila-Worley R., Ausubel F.M. (2012). Immune defense mechanisms in the *Caenorhabditis elegans* intestinal epithelium. Curr. Opin. Immunol..

[8] Montalvo-Katz S., Huang H., Appel M.D., Berg M., Shapira M. (2013). Association with soil bacteria enhances p38-dependent infection resistance in *Caenorhabditis elegans*. Infect. Immunity.

[9] Berg M., Stenuit B., Ho J., Wang A., Parke C., Knight M., Alvarez-Cohen L., Shapira M. (2016). Assembly of the *Caenorhabditis elegans* gut microbiota from diverse soil microbial environments. ISME J..

[10] Berg M., Zhou X.Y., Shapira M. (2016). Host-Specific Functional Significance of Caenorhabditis Gut Commensals. Front. Microbiol..

[11] Dirksen P., Marsh S.A., Braker I., Heitland N., Wagner S., Nakad R., Mader S., Petersen C., Kowallik V., Rosenstiel P. (2016). The native microbiome of the nematode *Caenorhabditis elegans*: Gateway to a new host-microbiome model. BMC Biol..

[12] Samuel B.S., Rowedder H., Braendle C., Felix M.A., Ruvkun G. (2016). *Caenorhabditis elegans* responses to bacteria from its natural habitats. Proc. Natl. Acad. Sci. USA.

[13] Rosshart S.P., Vassallo B.G., Angeletti D., Hutchinson D.S., Morgan A.P., Takeda K., Hickman H.D., McCulloch J.A., Badger J.H., Ajami N.J. (2017). Wild Mouse Gut Microbiota Promotes Host Fitness and Improves Disease Resistance. Cell.

[14] Cohen L.B., Troemel E.R. (2015). Microbial pathogenesis and host defense in the nematode *C. elegans*. Curr. Opin. Microbiol..

[15] Marsh E.K., May R.C. (2012). *Caenorhabditis elegans*, a model organism for investigating immunity. Appl. Environ. Microbiol..

[16] Engelmann I., Pujol N. (2010). Innate immunity in *C. elegans*. Adv. Exp. Med. Biol..

[17] Zhang R., Hou A. (2013). Host-Microbe Interactions in *Caenorhabditis elegans*. ISRN Microbiol..

[18] Ermolaeva M.A., Schumacher B. (2014). Insights from the worm: The *C. elegans* model for innate immunity. Semin. Immunol..

[19] Mahajan-Miklos S., Tan M.W., Rahme L.G., Ausubel F.M. (1999). Molecular mechanisms of bacterial virulence elucidated using a *Pseudomonas aeruginosa*-*Caenorhabditis elegans* pathogenesis model. Cell.

[20] Xu A., Shi G., Liu F., Ge B. (2013). *Caenorhabditis elegans* MOM-4 is required for the activation of the p38 MAPK signaling pathway in the response to *Pseudomonas aeruginosa* infection. Protein Cell.

[21] Powell J.R., Kim D.H., Ausubel F.M. (2009). The G protein-coupled receptor FSHR-1 is required for the *Caenorhabditis elegans* innate immune response. Proc. Natl. Acad. Sci. USA.

[22] Estes K.A., Dunbar T.L., Powell J.R., Ausubel F.M., Troemel E.R. (2010). bZIP transcription factor ZIP-2 mediates an early response to *Pseudomonas aeruginosa* infection in *Caenorhabditis elegans*. Proc. Natl. Acad. Sci. USA.

[23] Aballay A., Drenkard E., Hilbun L.R., Ausubel F.M. (2003). *Caenorhabditis elegans* innate immune response triggered by Salmonella enterica requires intact LPS and is mediated by a MAPK signaling pathway. Curr. Biol..

[24] Kurz C.L., Chauvet S., Andres E., Aurouze M., Vallet I., Michel G.P., Uh M., Celli J., Filloux A., De Bentzmann S. (2003). Virulence factors of the human opportunistic pathogen *Serratia marcescens* identified by in vivo screening. EMBO J..

[25] Sifri C.D., Begun J., Ausubel F.M., Calderwood S.B. (2003). *Caenorhabditis elegans* as a model host for Staphylococcus aureus pathogenesis. Infect. Immunity.

[26] Bae T., Banger A.K., Wallace A., Glass E.M., Aslund F., Schneewind O., Missiakas D.M. (2004). Staphylococcus aureus virulence genes identified by bursa aurealis mutagenesis and nematode killing. Proc. Natl. Acad. Sci. USA.

[27] Bolm M., Jansen W.T., Schnabel R., Chhatwal G.S. (2004). Hydrogen peroxide-mediated killing of *Caenorhabditis elegans*: A common feature of different streptococcal species. Infect. Immunity.

[28] Luallen R.J., Bakowski M.A., Troemel E.R. (2015). Characterization of microsporidia-induced developmental arrest and a transmembrane leucine-rich repeat protein in *Caenorhabditis elegans*. PLoS ONE.

[29] Bakowski M.A., Desjardins C.A., Smelkinson M.G., Dunbar T.A., Lopez-Moyado I.F., Rifkin S.A., Cuomo C.A., Troemel E.R. (2014). Ubiquitin-mediated response to microsporidia and virus infection in *C. elegans*. PLoS Pathog..

[30] Estes K.A., Szumowski S.C., Troemel E.R. (2011). Non-lytic, actin-based exit of intracellular parasites from *C. elegans* intestinal cells. PLoS Pathog..

[31] Pukkila-Worley R., Ausubel F.M., Mylonakis E. (2011). Candida albicans infection of *Caenorhabditis elegans* induces antifungal immune defenses. PLoS Pathog..

[32] Mylonakis E., Ausubel F.M., Perfect J.R., Heitman J., Calderwood S.B. (2002). Killing of *Caenorhabditis elegans* by Cryptococcus neoformans as a model of yeast pathogenesis. Proc. Natl. Acad. Sci. USA.

[33] Tang R.J., Breger J., Idnurm A., Gerik K.J., Lodge J.K., Heitman J., Calderwood S.B., Mylonakis E. (2005). Cryptococcus neoformans gene involved in mammalian pathogenesis identified by a *Caenorhabditis elegans* progeny-based approach. Infect. Immunity.

[34] Wilkins C., Dishongh R., Moore S.C., Whitt M.A., Chow M., Machaca K. (2005). RNA interference is an antiviral defence mechanism in *Caenorhabditis elegans*. Nature.

[35] Schott D.H., Cureton D.K., Whelan S.P., Hunter C.P. (2005). An antiviral role for the RNA interference machinery in *Caenorhabditis elegans*. Proc. Natl. Acad. Sci. USA.

[36] Gammon D.B., Ishidate T., Li L., Gu W., Silverman N., Mello C.C. (2017). The Antiviral RNA Interference Response Provides Resistance to Lethal Arbovirus Infection and Vertical Transmission in *Caenorhabditis elegans*. Curr. Biol. CB.

[37] Liu W.H., Lin Y.L., Wang J.P., Liou W., Hou R.F., Wu Y.C., Liao C.L. (2006). Restriction of vaccinia virus replication by a CED-3 and CED-4-dependent pathway in *Caenorhabditis elegans*. Proc. Natl. Acad. Sci. USA.

[38] Lu R., Maduro M., Li F., Li H.W., Broitman-Maduro G., Li W.X., Ding S.W. (2005). Animal virus replication and RNAi-mediated antiviral silencing in *Caenorhabditis elegans*. Nature.

[39] Chao J.A., Lee J.H., Chapados B.R., Debler E.W., Schneemann A., Williamson J.R. (2005). Dual modes of RNA-silencing suppression by Flock House virus protein B2. Nat. Struct. Mol. Biol..

[40] Lu R., Yigit E., Li W.X., Ding S.W. (2009). An RIG-I-Like RNA helicase mediates antiviral RNAi downstream of viral siRNA biogenesis in *Caenorhabditis elegans*. PLoS Pathog..

[41] Felix M.A., Ashe A., Piffaretti J., Wu G., Nuez I., Belicard T., Jiang Y., Zhao G., Franz C.J., Goldstein L.D. (2011). Natural and experimental infection of Caenorhabditis nematodes by novel viruses related to nodaviruses. PLoS Biol..

[42] Franz C.J., Zhao G., Felix M.A., Wang D. (2012). Complete genome sequence of Le Blanc virus, a third *Caenorhabditis nematode*-infecting virus. J. Virol..

[43] Franz C.J., Renshaw H., Frezal L., Jiang Y., Felix M.A., Wang D. (2014). Orsay, Santeuil and Le Blanc viruses primarily infect intestinal cells in Caenorhabditis nematodes. Virology.

[44] Jiang H., Franz C.J., Wu G., Renshaw H., Zhao G., Firth A.E., Wang D. (2014). Orsay virus utilizes ribosomal frameshifting to express a novel protein that is incorporated into virions. Virology.

[45] Ashe A., Belicard T., Le Pen J., Sarkies P., Frezal L., Lehrbach N.J., Felix M.A., Miska E.A. (2013). A deletion polymorphism in the *Caenorhabditis elegans* RIG-I homolog disables viral RNA dicing and antiviral immunity. eLife.

[46] Guo X., Zhang R., Wang J., Ding S.W., Lu R. (2013). Homologous RIG-I-like helicase proteins direct RNAi-mediated antiviral immunity in *C. elegans* by distinct mechanisms. Proc. Natl. Acad. Sci. USA.

[47] Guo X., Lu R. (2013). Characterization of virus-encoded RNAi suppressors in *Caenorhabditis elegans*. J. Virol..

[48] Le Pen J., Jiang H., Domenico T.D., Kneuss E., Kosalka J., Morgan M., Much M., Rudolph L.M.K., Enright A.J., O’Carroll D. (2017). Terminal uridylyltransferases target RNA viruses as part of the innate immune system in animals. bioRxiv.

[49] Sarkies P., Ashe A., Le Pen J., McKie M.A., Miska E.A. (2013). Competition between virus-derived and endogenous small RNAs regulates gene expression in *Caenorhabditis elegans*. Genome Res..

[50] Chen K., Franz C.J., Jiang H., Jiang Y., Wang D. (2017). An evolutionarily conserved transcriptional response to viral infection in Caenorhabditis nematodes. BMC Genom..

[51] Tanguy M., Veron L., Stempor P., Ahringer J., Sarkies P., Miska E.A. (2017). An Alternative STAT Signaling Pathway Acts in Viral Immunity in *Caenorhabditis elegans*. mBio.

[52] Jiang H., Chen K., Sandoval L.E., Leung C., Wang D. (2017). An Evolutionarily Conserved Pathway Essential for Orsay Virus Infection of *Caenorhabditis elegans*. mBio.

[53] Wei J.Z., Hale K., Carta L., Platzer E., Wong C., Fang S.C., Aroian R.V. (2003). *Bacillus thuringiensis* crystal proteins that target nematodes. Proc. Natl. Acad. Sci. USA.

[54] Los F.C., Kao C.Y., Smitham J., McDonald K.L., Ha C., Peixoto C.A., Aroian R.V. (2011). RAB-5- and RAB-11-dependent vesicle-trafficking pathways are required for plasma membrane repair after attack by bacterial pore-forming toxin. Cell Host Microbe.

[55] Spanier B., Starke M., Higel F., Scherer S., Fuchs T.M. (2010). Yersinia enterocolitica infection and tcaA-dependent killing of *Caenorhabditis elegans*. Appl. Environ. Microbiol..

[56] Kothe M., Antl M., Huber B., Stoecker K., Ebrecht D., Steinmetz I., Eberl L. (2003). Killing of *Caenorhabditis elegans* by Burkholderia cepacia is controlled by the CEP quorum-sensing system. Cell. Microbiol..

[57] Gan Y.H., Chua K.L., Chua H.H., Liu B., Hii C.S., Chong H.L., Tan P. (2002). Characterization of Burkholderia pseudomallei infection and identification of novel virulence factors using a *Caenorhabditis elegans* host system. Mol. Microbiol..

[58] Garsin D.A., Sifri C.D., Mylonakis E., Qin X., Singh K.V., Murray B.E., Calderwood S.B., Ausubel F.M. (2001). A simple model host for identifying Gram-positive virulence factors. Proc. Natl. Acad. Sci. USA.

[59] Thomsen L.E., Slutz S.S., Tan M.W., Ingmer H. (2006). *Caenorhabditis elegans* is a model host for Listeria monocytogenes. Appl. Environ. Microbiol..

[60] Pradel E., Zhang Y., Pujol N., Matsuyama T., Bargmann C.I., Ewbank J.J. (2007). Detection and avoidance of a natural product from the pathogenic bacterium Serratia marcescens by *Caenorhabditis elegans*. Proc. Natl. Acad. Sci. USA.

[61] Schulenburg H., Ewbank J.J. (2007). The genetics of pathogen avoidance in *Caenorhabditis elegans*. Mol. Microbiol..

[62] Schulenburg H., Kurz C.L., Ewbank J.J. (2004). Evolution of the innate immune system: The worm perspective. Immunol. Rev..

[63] Darby C. (2005). Interactions with microbial pathogens. WormBook: The Online Review of C. elegans Biology.

[64] Gordon S. (2002). Pattern recognition receptors: Doubling up for the innate immune response. Cell.

[65] Nicholas H.R., Hodgkin J. (2004). Responses to infection and possible recognition strategies in the innate immune system of *Caenorhabditis elegans*. Mol. Immunol..

[66] Rajamuthiah R., Mylonakis E. (2014). Effector triggered immunity. Virulence.

[67] Kim D.H., Feinbaum R., Alloing G., Emerson F.E., Garsin D.A., Inoue H., Tanaka-Hino M., Hisamoto N., Matsumoto K., Tan M.W. (2002). A conserved p38 MAP kinase pathway in *Caenorhabditis elegans* innate immunity. Science.

[68] Bolz D.D., Tenor J.L., Aballay A. (2010). A conserved PMK-1/p38 MAPK is required in *Caenorhabditis elegans* tissue-specific immune response to Yersinia pestis infection. J. Biol. Chem..

[69] Huffman D.L., Abrami L., Sasik R., Corbeil J., van der Goot F.G., Aroian R.V. (2004). Mitogen-activated protein kinase pathways defend against bacterial pore-forming toxins. Proc. Natl. Acad. Sci. USA.

[70] Troemel E.R., Chu S.W., Reinke V., Lee S.S., Ausubel F.M., Kim D.H. (2006). p38 MAPK regulates expression of immune response genes and contributes to longevity in *C. elegans*. PLoS Genetics.

[71] Evans E.A., Chen W.C., Tan M.W. (2008). The DAF-2 insulin-like signaling pathway independently regulates aging and immunity in *C. elegans*. Aging Cell.

[72] Pellegrino M.W., Nargund A.M., Kirienko N.V., Gillis R., Fiorese C.J., Haynes C.M. (2014). Mitochondrial UPR-regulated innate immunity provides resistance to pathogen infection. Nature.

[73] Singh V., Aballay A. (2009). Regulation of DAF-16-mediated Innate Immunity in *Caenorhabditis elegans*. J. Biol. Chem..

[74] Shapira M., Hamlin B.J., Rong J., Chen K., Ronen M., Tan M.W. (2006). A conserved role for a GATA transcription factor in regulating epithelial innate immune responses. Proc. Natl. Acad. Sci. USA.

[75] Visvikis O., Ihuegbu N., Labed S.A., Luhachack L.G., Alves A.F., Wollenberg A.C., Stuart L.M., Stormo G.D., Irazoqui J.E. (2014). Innate host defense requires TFEB-mediated transcription of cytoprotective and antimicrobial genes. Immunity.

[76] Jansson H.B. (1994). Adhesion of Conidia of Drechmeria coniospora to *Caenorhabditis elegans* Wild Type and Mutants. J. Nematol..

[77] Zugasti O., Bose N., Squiban B., Belougne J., Kurz C.L., Schroeder F.C., Pujol N., Ewbank J.J. (2014). Activation of a G protein-coupled receptor by its endogenous ligand triggers the innate immune response of *Caenorhabditis elegans*. Nat. Immunol..

[78] Diogo J., Bratanich A. (2014). The nematode *Caenorhabditis elegans* as a model to study viruses. Arch. Virol..

[79] Gammon D.B. (2017). *Caenorhabditis elegans* as an Emerging Model for Virus-Host Interactions. J. Virol..

[80] Christensen M., Estevez A., Yin X., Fox R., Morrison R., McDonnell M., Gleason C., Miller D.M., Strange K. (2002). A primary culture system for functional analysis of *C. elegans* neurons and muscle cells. Neuron.

[81] Zhang S., Banerjee D., Kuhn J.R. (2011). Isolation and culture of larval cells from *C. elegans*. PLoS ONE.

[82] Leyva-Diaz E., Stefanakis N., Carrera I., Glenwinkel L., Wang G., Driscoll M., Hobert O. (2017). Silencing of Repetitive DNA Is Controlled by a Member of an Unusual *Caenorhabditis elegans* Gene Family. Genetics.

[83] Thomas J.H. (2006). Adaptive evolution in two large families of ubiquitin-ligase adapters in nematodes and plants. Genome Res..

[84] Reddy K.C., Dror T., Sowa J.N., Panek J., Chen K., Lim E.S., Wang D., Troemel E.R. (2017). An Intracellular Pathogen Response Pathway Promotes Proteostasis in *C. elegans*. Curr. Biol. CB.

[85] Jose A.M., Kim Y.A., Leal-Ekman S., Hunter C.P. (2012). Conserved tyrosine kinase promotes the import of silencing RNA into *Caenorhabditis elegans* cells. Proc. Natl. Acad. Sci. USA.

[86] Tampakakis E., Peleg A.Y., Mylonakis E. (2009). Interaction of Candida albicans with an intestinal pathogen, Salmonella enterica serovar Typhimurium. Eukaryot. Cell.

[87] Peleg A.Y., Tampakakis E., Fuchs B.B., Eliopoulos G.M., Moellering R.C., Mylonakis E. (2008). Prokaryote-eukaryote interactions identified by using *Caenorhabditis elegans*. Proc. Natl. Acad. Sci. USA.

